# The burden of hypertension in sub-Saharan Africa: a four-country cross sectional study

**DOI:** 10.1186/s12889-015-2546-z

**Published:** 2015-12-05

**Authors:** David Guwatudde, Joan Nankya-Mutyoba, Robert Kalyesubula, Carien Laurence, Clement Adebamowo, IkeOluwapo Ajayi, Francis Bajunirwe, Marina Njelekela, Faraja S. Chiwanga, Todd Reid, Jimmy Volmink, Hans-Olov Adami, Michelle D. Holmes, Shona Dalal

**Affiliations:** Department of Epidemiology and Biostatistics, School of Public Health, Makerere University College of Health Sciences, Kampala, Uganda; Department of Medicine, School of Medicine, Makerere University College of Health Sciences, Kampala, Uganda; Centre for Evidence-based Health Care, Faculty of Medicine and Health Sciences, Stellenbosch University, Cape Town, South Africa; Institute of Human Virology, Abuja, Nigeria; Greenbaum Cancer Center and Institute of Human Virology, University of Maryland, School of Medicine, Baltimore, MD USA; Department of Epidemiology and Medical Statistics, Faculty of Public Health, College of Medicine, University of Ibadan, Ibadan, Nigeria; Department of Community Health, Mbarara University Of Science and Technology, Mbarara, Uganda; Department of Physiology, Muhimbili University of Health and Allied Sciences, Dar es Salaam, Tanzania; Department of Epidemiology, Harvard School of Public Health, Boston, MA USA; The South African Cochrane Centre, South African Medical Research Council, Cape Town, South Africa; Department of Medical Epidemiology and Biostatistics, Karolinska Institute, Stockholm, Sweden; Channing Division of Network Medicine, Department of Medicine, Brigham and Women’s Hospital and Harvard Medical School, Boston, MA USA

**Keywords:** Prevalence of hypertension, Prevalence of pre-hypertension, Sub-Saharan Africa, Risk factors for hypertension, Chronic disease epidemiology

## Abstract

**Background:**

Hypertension, the leading single cause of morbidity and mortality worldwide, is a growing public health problem in sub-Saharan Africa (SSA). Few studies have estimated and compared the burden of hypertension across different SSA populations. We conducted a cross-sectional analysis of blood pressure data collected through a cohort study in four SSA countries, to estimate the prevalence of pre-hypertension, the prevalence of hypertension, and to identify the factors associated with hypertension.

**Methods:**

Participants were from five different population groups defined by occupation and degree of urbanization, including rural and peri-urban residents in Uganda, school teachers in South Africa and Tanzania, and nurses in Nigeria. We used a standardized questionnaire to collect data on demographic and behavioral characteristics, injuries, and history of diagnoses of chronic diseases and mental health. We also made physical measurements (weight, height and blood pressure), as well as biochemical measurements; which followed standardized protocols across the country sites. Modified Poison regression modelling was used to estimate prevalence ratios (PR) as measures of association between potential risk factors and hypertension.

**Results:**

The overall age-standardized prevalence of hypertension among the 1216 participants was 25.9 %. Prevalence was highest among nurses with an age-standardized prevalence (ASP) of 25.8 %, followed by school teachers (ASP = 23.2 %), peri-urban residents (ASP = 20.5 %) and lowest among rural residents (ASP = 8.7 %). Only 50.0 % of participants with hypertension were aware of their raised blood pressure. The overall age-standardized prevalence of pre-hypertension was 21.0 %. Factors found to be associated with hypertension were: population group, older age, higher body mass index, higher fasting plasma glucose level, lower level of education, and tobacco use.

**Conclusions:**

The prevalence of hypertension and pre-hypertension are high, and differ by population group defined by occupation and degree of urbanization. Only half of the populations with hypertension are aware of their hypertension, indicating a high burden of undiagnosed and un-controlled high blood pressure in these populations.

## Background

Hypertension, the most common cardiovascular disorder affecting approximately one billion people globally, remains the leading single contributor to global burden of disease and mortality accounting for approximately 9.4 million deaths annually [1–5]. In 2000, there were an estimated 972 million people with hypertension, 65 % of whom lived in the developing world, with the number predicted to grow to 1.5 billion by 2025. The effects of hypertension if not controlled are devastating, and may include stroke, myocardial infarction, cardiac failure, and renal failure among others [6].

A number of studies indicate that hypertension in sub-Sahara Africa (SSA) is a widespread problem, and in some communities it has been reported to be as high as 38 % [4, 7–9]. It is estimated that out of the approximately 650 million people in SSA, between 10 to 20 million may have hypertension [7]. However many countries in SSA still lack detailed basic data on the prevalence of hypertension [3], and how this is distributed in the different SSA populations. Further, few studies have reported on the proportion of persons with hypertension who are aware of their hypertension, and the burden of pre-hypertension. We analyzed data collected through a cohort study conducted by the Partnership for Cohort Research and Training (PaCT) consortium, in four sub-Saharan countries including Nigeria, South Africa, Tanzania and Uganda [10]; to estimate the prevalence of hypertension and pre-hypertension, and to identify factors associated with hypertension.

## Methods

A detailed description of methods used in the parent PaCT study from which this analysis is derived has been reported elsewhere [10]. Here we only describe methods relevant to results presented in this article.

### Study design

The parent study was conducted using a cohort study design between January 2011 and July 2012. Participants were followed for up to six months, with evaluations at baseline, and at six months only. Results presented in this article are a cross-sectional analysis of the baseline data.

### Study population

The study was conducted in four sub-Saharan countries, including Tanzania, South Africa, Uganda and Nigeria. Participants were enrolled from 5 different population groups, three defined by occupation, and two by degree of urbanization. Tanzania and South Africa enrolled school teachers, Nigeria enrolled nurses, whereas Uganda enrolled rural and peri-urban residents. Eligible subjects were adults aged 18 years or older, with no intension of migrating outside of their community of residence within the next 6 months (in Uganda), or retiring from service in the next 6 months (for Tanzania, South Africa and Nigeria).

School teachers in Tanzania were enrolled from 18 randomly selected public schools in Dar es Salaam. In South Africa, school teachers were enrolled from government schools in Cape Town Metropolitan area, where 111 schools with 20 or more teachers were invited to participate in the study. In Nigeria, a random sample of nurses were enrolled from two urban hospitals; one located in Abuja city and another in a semi-urban setting 1.5 h outside of Abuja city. In Uganda, participants were enrolled from two geographic locations; a peri-urban community in the Wakiso District 10 miles north of Kampala city, and from a rural community in Bushenyi District 200 miles west of Kampala city. In Wakiso District, a random sample of households was selected from two parishes comprising 13 villages. In Bushenyi District, households were randomly selected from an enumerated list of all the households in each village.

### Ethics, consent and permissions

Informed consent was obtained from each subject either by voluntarily posting back a signed form with a completed questionnaire (South Africa and Tanzania) or through documentation with trained interviewers (Nigeria and Uganda). The pilot studies were approved by the: a) Harvard School of Public Health Institutional Review Board, b) Institute of Human Virology Heath Research Ethics Committee in Nigeria; c) Health Research Ethics Committee of the Faculty of Health Sciences, Stellenbosch University in South Africa; d) National Institute for Medical Research in Tanzania; and in Uganda; e) Makerere University School of Public Health Higher Degrees, Research and Ethics Committee; f) Mbarara University of Science and Technology Ethics Committee, and g) the Uganda National Council of Science and Technology.

### Measurements

We used a standardized questionnaire to collect data on socio-economic characteristics, history of infectious and chronic disease diagnoses, mental health, injuries, and common risk factors for non-communicable diseases (NCDs) including tobacco use, alcohol use, and physical activity. Most of the questions were adapted from the World Health Organization STEPS instrument developed for use in resource-limited countries [11]. Physical and biochemical measurements were also made following a standardized protocol across the five study sites. Physical measurements included weight, height, and blood pressure. Height was measured without shoes, to the nearest centimetre. Weight was measured with the participants in light clothing and without footwear using a weighing scale to the nearest tenth of a kilogram. Blood pressure measurements were taken on the left arm with the participant in the sitting position using a cali-brated electronic blood pressure device (Welch-Allyn®). Three systolic and diastolic blood pressure measurements were taken at least five minutes apart. The average of the last two blood pressure readings was used in this analysis.

After administering the questionnaire, interviewers requested participants for an appointment to return the following morning so that a blood sample could be obtained to measure plasma glucose levels. Participants were instructed to fast overnight and no exercise or smoking in the morning, in preparation for obtaining a blood sample to conduct a fasting plasma glucose (FPG) measurements. The following morning the interviewer returned and collected the participant’s finger prick blood sample for an FPG test using a digital glucometer (On-Call® Plus, ACON Laboratories). Only participants reporting compliance with an overnight 8-h fast, no exercise or smoking that morning were eligible for the finger prick blood sample collection. Non-compliers were rescheduled to a future date where possible; otherwise this procedure was omitted in such participants. FPG levels were recorded in milli moles per liter (mmol/L).

### Statistical analysis

Participants were classified in different blood pressure categories based on the World Health Organization (WHO) cut-off criteria [12, 13]. Thus a participant was classified as being hypertensive if their average systolic blood pressure (SBP) was at least 140 mm Hg, and/or their average diastolic blood pressure (DBP) was at least 90 mm Hg, or if they reported being on regular anti-hypertensive therapy. A participant was classified as being pre-hypertensive if their average SBP was between 120 and 139 mm Hg (inclusive), and/or their DBP was between 80 and 89 mm Hg (inclusive), and not on any anti-hypertensive therapy.

The prevalence of pre-hypertension and hypertension were calculated as the percentage of participants classified as being pre-hypertensive or hypertensive, respectively. To enable comparison of prevalence across the five population groups with differing age-structures, prevalence was directly age-standardized to the World Health Organization’s 2000–2025 world standard population age structure [14, 15].

Body Mass Index (BMI) was computed by dividing the weight (kg) by the height in meters squared (m^2^) and used to develop the categories of: underweight (less or equal to 18.5), normal weight (18.5–24.9), overweight (25.0–29.9), and obese (30 or higher). Participants were also categorized on the basis of their plasma glucose levels status using the WHO definition and diagnosis of diabetes mellitus criteria as follows: Normal (less than 6.1 mmol/L), Pre-diabetes (6.1 to 6.9 mmol/L), and diabetes or probable diabetes (greater than 6.9 mmol/L, or currently on anti-diabetes mellitus medication) [16].

To identify factors associated with hypertension, the modified Poisson regression model with robust variance was used to estimate both the crude and adjusted prevalence ratios (PR) [17, 18], with their corresponding 95 % confidence intervals (95 % CI). The modified Poisson regression model, a model that uses a robust error variance, was preferred to avoid under estimation of the standard errors for the estimated risk ratios that is usually the case with logistic regression modelling when the prevalence of the outcome is greater than 10 % [17, 18]. Potential confounding and interaction variables that were assessed for inclusion in the model were: age, sex, level of education, body mass index, population group, FPG category, family history of hypertension (first degree relatives only), whether the participant met the WHO physical activity recommendations [19], and tobacco use category. A step-by-step backward elimination of these variables from the model was used to identify those to be retained in the model. The criterion used to retain variables in the model were if a variable was significantly associated with hypertension using a 5 % level of statistical significance (α = 0.05), or if addition of the variable in the model led to a change of at least 10 % in any of the significant PR estimates of any variable already in the model. Cases with missing observations were excluding from the model on a case by case basis. All statistical analyses were performed using STATA version 13 (StataCorp, College Station, Texas, USA).

## Results

### Study participants

A total of 1414 subjects participated in the baseline cohort pilot study. Of the 1414 participants, at least two blood pressure measurements were available from 1269 (89.7 %) participants and were used in this analysis. These included 163 nurses in Nigeria, 477 school teachers in South Africa, 167 school teachers in Tanzania, and from Uganda 297 peri-urban and 165 rural residents.

Of the 1269 participants, 820 (64.6 %) were females, 671 (52.9 %) were aged 30 to 49 years, and 879 (69.3 %) had received up to secondary school education or at least 8 years of schooling. The majority of participants (772 or 60.8 %) were over-weight with a body mass index (BMI) of 25 kilograms per squared meter (kg/m^2^) or higher, including the 407 (32.1 %) that were obese (BMI ≥ 30 kg/m^2^). Of the participants, 121 (9.5 %) reported current use of some form of tobacco and only 272 (21.4 %) reported physical activity levels that meet the WHO recommendation of at least 75 min of vigorous-intensity or 150 min of moderate-intensity physical activity per week [19]. The rest of characteristics of the participants are presented in Table 1.

**Table 1:** Participant characteristics

Characteristic		- n -	Percentage
Population group	Rural residents	165	13.0 %
	Peri-urban residents	297	23.4 %
	School teachers, Tanzania	167	13.2 %
	Nurses, Nigeria	163	12.8 %
	School teachers, South Africa	477	37.6 %
Sex:	Males	449	35.4 %
	Females	820	64.6 %
Age in years:	18–29	231	18.2 %
	30–39	287	22.6 %
	40–49	384	30.3 %
	≥50	314	24.7 %
	Not stated	53	4.2 %
Level of education:	None	49	3.9 %
	Primary not completed (1–6 yrs)	142	11.2 %
	Primary completed (7 yrs)	87	6.9 %
	Secondary/High Sch (8–13 yrs)	406	31.9 %
	University	516	40.6 %
	Not stated	71	5.6 %
Marital status:	Never married	436	34.4 %
	Married/Co-habiting	655	51.6 %
	Separated/divorced	100	7.9 %
	Other	51	4.0 %
	Not stated	27	2.1 %
Family history of HPT^a^	No	869	68.5 %
	Yes	400	31.5 %
Body mass index (kg/m^2^)	<18.5	23	1.8 %
	18.5–24.9	386	30.4 %
	25.0–29.9	365	28.8 %
	≥30.0	407	32.1 %
	Not stated	71	5.6 %
Tobacco use type:	Never	842	66.4 %
	Stopped	104	8.2 %
	Current user	121	9.5 %
	Not stated	202	15.9 %

^a^ *HPT* Hypertension

### Prevalence of hypertension and pre-hypertension

Of the 1269 participants, 468 were classified as being hypertensive giving an overall crude prevalence of 36.9 %. The overall age-adjusted prevalence was 25.9 %. Hypertension was highest amongst nurses in Nigeria with age-adjusted prevalence of 25.8 %, followed by school teachers in South Africa with age-adjusted prevalence of 23.2 %, then by school teachers in Tanzania with age-adjusted prevalence of 23.1 %, whereas the peri-urban and rural residents in Uganda had an age-adjusted prevalence of 20.5 % and 8.7 %, respectively (Table 2).

**Table 2:** Prevalence of hypertension and pre-hypertension by population group

Characteristic		Hypertension		Pre-hypertension	
	- n -	Crude	Age-standardized	Crude	Age-standardized
All participants	1269	468 (36.8 %)	25.9 %	378 (29.8 %)	21.0 %
Rural residents, Uganda	165	21 (12.7 %)	8.7 %	55 (33.3 %)	19.5 %
Peri-urban residents, Uganda	297	70 (23.6 %)	20.5 %	97 (32.7 %)	21.6 %
Nurses, Nigeria	163	77 (47.2 %)	25.8 %	34 (20.9 %)	16.3 %
School teachers, South Africa	477	228 (47.8 %)	23.2 %	166 (34.8 %)	26.0 %
School teachers, Tanzania	167	72 (43.1 %)	23.1 %	26 (15.6 %)	7.5 %

**Fig. 1:**
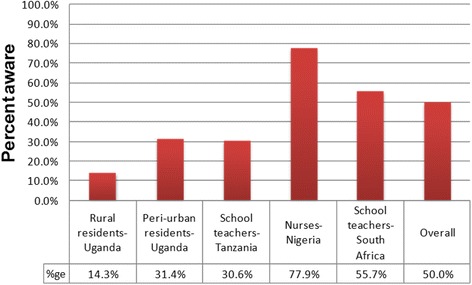
Percentage of participants with hypertension who are aware of their high blood pressure

Only 234 of the 468 participants with hypertension (50.0 %), were aware of their raised blood pressure condition. The level of awareness varied significantly by population group (*p* < 0.001), being highest among nurses in Nigeria at 77.9 %, and lowest among rural residents in Uganda at 14.3 %. Figure 1 shows the percentage of participants with hypertension who were aware of their raised blood pressure status, by population group.

A total of 378 participants were classified as being pre-hypertensive, giving a crude prevalence of 29.8 %, and an overall age-adjusted prevalence of 21.0 %. Pre-hypertension was highest among school teachers in South Africa with an age-adjusted prevalence of 26.0 %, followed by peri-urban and rural residents in Uganda with age-adjusted prevalence of 21.6 % and 19.5 % respectively, followed by nurses in Nigeria and school teachers in Tanzania with age-adjusted prevalence of 16.3 % and 7.5 %, respectively (Table 2).

### Factors associated with hypertension

Factors found to be associated with hypertension included population group, age, body mass index, fasting plasma glucose (FPG), level of education, and tobacco use. Compared to the rural residents in Uganda, the prevalence of hypertension was highest among nurses in Nigeria with an adjusted prevalence ratio (PR) of 6.49 [95 % CI = 2.26 – 18.7]. The prevalence of hypertension was significantly higher among participants aged 50 years or older compared to those aged 18 – 29 years with an adjusted PR of 2.20 [95 % CI = 1.49 – 3.25]. We found a significantly lower adjusted prevalence among participants who attained secondary school level of education compared to those reporting no formal education, with a PR of 0.54 [95 % CI = 0.31 – 0.93]. We also the prevalence of hypertension to be significantly higher among participants with BMI between 25 to 29.9 kg/m^2^ compared to those with BMI less than 25, with an adjusted PR = 1.61 [95 % CI = 1.18 – 2.20], and even higher among the obese (BMI ≥ 30 kg/m^2^) participants compared to those with BMI less than 25 with an adjusted PR = 2.11 [95 % CI = 1.56 – 2.84]. The prevalence of hypertension was also more likely to be higher among participants with fasting plasma glucose (FPG) of 7.0 mmol/L or higher, compared to those with FPG less than 6.1 mmol/L, with an adjusted PR of 1.38 [95 % CI = 1.10 – 1 171]. Finally, the prevalence of hypertension was significantly higher among participants reporting to use unfiltered tobacco, compared to those reporting to had never used tobacco, with an adjusted PR of 1.7 [1.04 – 2.91] (Table 3).

**Table 3:** Crude and adjusted prevalence ratio (PR) estimates for hypertension

Variable		- n -	# Hypertensive (%)	Crude PR [95 % CI]	Adjusted PR^b^ [95 % CI]
Population group:					
	Rural residents	165	21 (12.7 %)	1.0	1.0
	Peri-urban residents	298	70 (23.5 %)	1.85 [1.18–2.90]	2.28 [1.38–3.76]
	School teachers, Tanzania	168	72 (42.9 %)	3.39 [2.19–5.24]	3.96 [2.22–7.06]
	Nurses, Nigeria	163	77 (47.2 %)	3.71 [2.41–5.71]	6.49 [2.26–18.7]
	School teachers, South Africa	478	229 (47.9 %)	3.76 [2.49–5.66]	2.91 [1.56–5.45]
Sex:					
	Males	451	146 (32.4 %)	1.0	1.0
	Females	821	323 (39.3 %)	1.21 [1.04–1.43]	0.94 [0.76–1.17]
Age (years):					
	18–29	215	40 (18.6 %)	1.0	1.0
	30–39	294	80 (27.2 %)	1.53 [1.10–2.13]	1.00 [0.65–1.53]
	40–49	385	147 (38.2 %)	2.11 [1.56–2.85]	1.28 [0.84–1.95]
	≥50	319	186 (58.3 %)	3.22 [2.41–4.30]	2.20 [1.49–3.25]
Level of education					
	None	49	15 (30.6 %)	1.0	1.0
	Primary not completed	143	28 (19.6 %)	0.64 [0.38–1.10]	0.81 [0.46–1.43]
	Primary completed	87	21 (24.1 %)	0.79 [0.45–1.38]	0.62 [0.33–1.17]
	Secondary school	406	137 (33.7 %)	1.10 [0.71–1.72]	0.54 [0.31–0.93]
	University or higher	516	229 (44.4 %)	1.46 [0.93–2.21]	0.56 [0.30–1.05]
Family history of hypertension:					
	No	871	290 (33.3 %)	1.0	1.0
	Yes	401	179 (44.6 %)	1.3 [1.15–1.54]	1.21 [0.96–1.52]
Body mass index (kg/m^2^):					
	<25.0	409	83 (20.3 %)	1.0	1.0
	25.0–29.9	367	131 (35.7 %)	1.78 [1.40–2.25]	1.61 [1.18–2.20]
	≥30.0	407	210 (51.6 %)	2.59 [2.09–3.21]	2.11 [1.56–2.84]
FPG^c^/DM^d^ category:					
	<6.1	603	168 (27.9 %)	1.0	1.0
	6.1–6.9	103	28 (27.2 %)	0.92 [0.66–1.27]	0.91 [0.64–1.30]
	≥7.0, or on DM^d^ Rx	135	80 (59.3 %)	1.94 [1.62–2.31]	1.38 [1.10–1.71]
Met WHO^e^ physical activity recommednations?					
	No			1.0	1.0
	Yes			0.60 [0.48–0.75]	2.48 [0.34–18.0]
Tobacco used:					
	Never	845	274 (32.4 %)	1.0	1.0
	Stopped	104	42 (40.4 %)	1.25 [0.97–1.60]	0.98 [0.73–1.31]
	Filtered	98	45 (45.9 %)	1.42 [1.12–1.79]	1.21 [0.91–1.60]
	Unfiltered	23	8 (34.8)	1.07 [0.61–1.89]	1.74 [1.04–2.91]

^b^Adjusted for Population group, Age, Level of education, BMI, FPG and Tobacco use

^c^ *FPG* Fasting Plasma Glucose (mmol/L)

^d^ *DM* Diabetes Mellitus

^e^ *WHO* World Health Organization

## Discussion

In this study we found the overall age-standardized prevalence of hypertension to be high in the population groups studied, at nearly 26 %. Prevalence varied significantly by population group defined by occupation, and degree of urbanization, from rural to peri-urban residents, and between school teachers and nurses that were from urban centers. Our findings clearly demonstrate the urban–rural divide of the burden of hypertension in sub-Saharan Africa that has been highlighted by others [3, 7, 20]. Surprising was the finding that prevalence was highest among the health care workers - nurses in Nigeria. The astoundingly high prevalence of hypertension signals an enormous future burden of hypertension-related NCDs. The variation by population groups also suggests that it might be more cost effective to design interventions that target specific population groups with higher burden of hypertension. Thus larger and more inclusive studies are needed to identify population groups with higher burden of hypertension.

We also found that overall, only 50 % of persons with hypertension were aware of their hypertension, demonstrating the high burden of undiagnosed and untreated hypertension in SSA, a problem that has been highlighted by others [3, 4, 21–23]. The complications of uncontrolled blood pressure are devastating and include a number of hypertension-related NCDs like myocardial infarction, stroke, chronic kidney disease, and renal failure all of which may be life threatening [24]. The high burden of undiagnosed and untreated hypertension also signals the need to increase detection rates of existing hypertension and provide resources for adequate treatment [4, 23].

We also found a high age-standardized prevalence of pre-hypertension at 21 %, which also varied significantly by population group. It is recognised that persons with pre-hypertension are at higher risk of progressing to hypertension, and becoming prone to hypertension-associated cardiovascular diseases [12, 25, 26]. In fact Vasan et al. (2001) estimated a 30 % conversion rate from pre-hypertension state to hypertension every four years [27]. Risk reduction interventions aimed at preventing persons with pre-hypertension progressing to hypertension are urgently needed to contribute to the overall reduction in the prevalence of hypertension in SSA.

The factors we found to be associated with hypertension in this study included population group, age, BMI, level of education, and tobacco use. Most of these have previously been identified some of the important risk factors for hypertension by the different studies conducted in different parts of SSA [3, 28–31]. However we recognize that perhaps because of the small sample size in our study, we were unable to disentangle the various determinants of hypertension in the population groups studied that may include urban/rural, high/low social economic status as defined by occupation, education, and country-specific gross level of income, and potential regional differences due to ethnicity/genetics, and other shared but unmeasured risk factors that may include stress, diet, etc. Thus substantial variation in the prevalence of hypertension remained in the multi-variable analysis. This variation seems to have at least two important consequences. The first is that it would be inconsiderate to make estimates for SSA based on small and arbitrary selected samples. Thus our findings can only be generalized to similar population groups included in our analysis, not to the whole SSA populations. Secondly, the fact that a six-fold variation for one of the population groups remained even in the multi-variable analysis (nurses in Nigeria), indicates that there must be other risk factors of fundamental importance for hypertension other than those analyzed here. Thus larger investigative studies of the type that PaCT represents are needed in SSA to tease these out.

## Conclusions

There are three key messages from this analysis:

- The prevalence of hypertension is high at 25.9 %, but differed significantly by population group defined by occupation and degree of urbanization. Interventions prioritizing higher prevalence population groups might be more effective in controlling hypertension in the population.
- Fifty percent of persons with hypertension are unaware of their hypertension, demonstrating a high burden of undiagnosed and uncontrolled hypertension, and signalling the need to increase detection rates of existing hypertension and provide resources for adequate treatment
- Pre-hypertension is also high at 21 %, an indicator of an enormous future burden of hypertension-related non-communicable diseases (NCDs) if no appropriate interventions are implemented. Interventions aimed at preventing persons with pre-hypertension progressing to hypertension are urgently needed.

## Abbreviations

ASP: Age-standardized prevalence
BMI: Body mass index
CI: Confidence interval
DBP: Diastolic blood pressure
DM: Diabetes mellitus
FPG: Fasting plasma glucose
HPT: Hypertension
kg/m^2^: Kilograms per squared meter
mm Hg: Millimetres of mercury
mmol/L: Milli moles per liter
NCD: Non-communicable diseases
PaCT: Partnership for cohort research and training
PRR: Prevalence rate ratio
SPB: Systolic blood pressure
SSA: Sub-Saharan Africa
WHO: World Health Organization
